# A novel virus-inducible enhancer of the *interferon-β* gene with tightly linked promoter and enhancer activities

**DOI:** 10.1093/nar/gku1018

**Published:** 2014-10-27

**Authors:** A. Raja Banerjee, Yoon Jung Kim, Tae Hoon Kim

**Affiliations:** 1 Department of Genetics, Yale University, School of Medicine, New Haven, CT 06520, USA; 2 Department of Biological Sciences, The University of Texas at Dallas, Richardson, TX 75080, USA

## Abstract

Long-range enhancers of transcription are a key component of the genomic regulatory architecture. Recent studies have identified bi-directionally transcribed RNAs emanating from these enhancers known as eRNAs. However, it remains unclear how tightly coupled eRNA production is with enhancer activity. Through our systematic search for long-range elements that interact with the interferon-β gene, a model system for studying inducible transcription, we have identified a novel enhancer, which we have named L2 that regulates the expression of interferon-β. We have demonstrated its virus-inducible enhancer activity by analyzing epigenomic profiles, transcription factor association, nascent RNA production and activity in reporter assays. This enhancer exhibits intimately linked virus-inducible enhancer and bidirectional promoter activity that is largely dependent on a conserved Interferon Stimulated Response Element and robustly generates virus inducible eRNAs. Notably, its enhancer and promoter activities are fully retained in reporter assays even upon a complete elimination of its associated eRNA sequences. Finally, we show that L2 regulates IFNB1 expression by siRNA knockdown of eRNAs, and the deletion of L2 in a BAC transfection assay. Thus, L2 is a novel enhancer that regulates IFNB1 and whose eRNAs exert significant activity *in vivo* that is distinct from those activities recapitulated in the luciferase reporter assays.

## INTRODUCTION

Transcriptional enhancers stimulate transcription rate or transcription probability of their target genes (1,2) and can do so from distances of over a megabase (Mb) (3). They exert very specific effects on developmental patterning (3,4), are involved in human disease (5) and are broadly reflective of cell-type-specific gene expression (6,7). These functional properties make them an essential component of the genomic regulatory architecture.

There are a number of notable sequence and epigenetic features that distinguish enhancers from the rest of the genome. First, enhancer sequences are highly conserved across species. Sequence conservation alone within the noncoding sequences has been used for genome-wide identification of novel enhancers (8,9). Second, enhancers function through their interaction with transcription regulatory factors, and as a consequence, exhibit DNase I hypersensitivity *in vivo* (10). The enhancer-associated transcription regulators mediate deposition of monomethylation of Lysine 4 of Histone 3 (H3K4me1) (11) and acetylation of Lysine 27 of Histone 3 (H3K27ac) (12) on the nearby nucleosomes, which define an epigenetic signature of enhancers that distinguish them from promoters (13,14). Third, enhancers most likely exert their effect on their target promoters by forming chromosomal loops and physically interacting with the distant target promoters (15). Cohesin, a chromosomal looping factor, has been implicated in maintaining these enhancer-promoter interactions (16,17). Fourth, bi-directional transcription of short, non-polyadenylated RNAs emanates from many enhancers (18–22). These enhancer-associated RNAs, or ‘eRNAs,’ have been implicated in chromatin remodeling and looping (21–24). However, the functional relationships of these various characteristics remain unclear.

The interferon-β gene (IFNB1) has long been a model system for studying inducible transcription (25–28), and is a critical component of the innate immune response. Viral infection activates a signaling cascade that culminates with the phosphorylation and subsequent nuclear translocation and DNA-binding of Interferon Response Factor 3 (IRF3), which activates IFNB1 transcription (29). The secreted IFNβ protein then binds to the Interferon receptor and activates interferon-stimulated genes (ISGs) through interferon-stimulated gene factor-3 (ISGF3) to generate an anti-viral cellular state (30).

As a part of the Type I interferon family of genes, IFNB1 is located on the short arm of chromosome 9, clustered with other Type I interferon genes (31). Accumulated work over several decades on the proximal 110 bp immediately upstream of the IFNB1 gene defined the original ‘enhanceosome’—a model of cooperative and synergistic activity of sequence-specific transcription factors leading to coactivator recruitment, chromatin modification and transcriptional activation (32). Previous work has implicated the existence of potential regulatory elements in addition to the proximal 110 bp that may be required for precise control of the activation of the IFNB1 promoter (33,34). Indeed, even a surrounding 34 kb of genomic sequence was still insufficient to match native IFNβ production on a per-copy basis, suggesting that potential regulatory elements exist outside that window (35,36). Alu repeat elements have been suggested to regulate IFNB1 by delivering NFκB *in trans* (37). However, no additional distal *cis*-regulatory elements have been described. Identification of relevant distal enhancers that regulate the IFNB1 gene will provide critical insights into long-range gene regulation that can be further dissected mechanistically based on the already formidable knowledge accumulated for the proximal enhancer.

In this work, we performed a combination of Chromosome Conformation Capture (3C), chromatin state assessments and sequence analyses to identify novel putative enhancers associated with the IFNB1. Using luciferase assays, we confirmed that a transcriptional element we named ‘L2′ is a virus inducible enhancer that can regulate transcription driven by IFNB1′s proximal regulatory elements. These experiments also showed that L2 includes bidirectional promoter activity that is intimately linked with its enhancer activity. We used global run-on sequencing (GRO-seq) analysis to map eRNA production, and used luciferase assays to show that L2 retained both its promoter and enhancer functions in the absence of its associated eRNA sequences. We conclude that L2 is a novel IFNB1-associated enhancer that exhibits promoter activity that is tightly linked to its enhancer activity. Notably, the specific sequences of its associated eRNAs are dispensable in reconstituting both L2′s promoter and enhancer activities in a heterologous reporter system. This suggests that this enhancer is a complex element that may exert multiple activities that stimulate transcription.

## MATERIALS AND METHODS

### Cells and treatment

Cell lines were purchased from American Type Culture Collection and cell culture media and supplements were purchased from Life Technologies, Inc., Carlsbad, CA. IMR90 primary human lung fibroblast cells were grown in Minimum Essential Media (MEM) supplemented with Earle's salt, 10% Fetal Bovine Serum (FBS) and 1-mM Sodium Pyruvate. HEK293 cells and NIH3T3 cells were each grown in Dulbecco's modified Eagle's medium supplemented with 10% FBS. Both were grown in 37°C incubator with 5% CO2.

Transfections used Lipofectamine 2000 (Life Technologies), preparing it with the DNA in Optimem (Life Technologies) per manufacturer's recommendations. The DNA–lipofectamine complexes were added to cells resuspended in growth media before plating. Transfected cells were placed back in the incubator for 3–5 h after transfection before any further treatment.

Sendai Virus (SeV) (Charles River Laboratories), was centrifuged to clarify the virus and stored at –80°C. For virus infections, we used 50 μl of virus per 1 ml of culture media. Interferon-β (Peprotech, catalog # 300–02BC) was used at 0.1 ng of IFN-β per 1 ml of culture media. These treatments were for 6 h, except for the Luciferase assays, which were for 24 h before prepping the cells for the assay.

### Chromosome conformation capture

3C experiments were performed as previously (38), but adjusted for scale and for use of EcoRI as the restriction enzyme (New England Biolabs, Ipswich, MA, USA). One hundred and seventy-two primer pairs anchored at IFNB1 (primer 23) covered hg18 chr9:20,996,400–22,347,415 (see Supplementary Table S1 for details). Large-scale 3C experiments were performed using 5 × 10^6^ IMR90 cells and 1.2 × 10^6^ nuclei per ligation. Small-scale 3C was performed using 1 × 10^6^ cells at large-scale volumes through formaldehyde crosslinking and quenching, reduced to cell-proportional scale from the cell lysis and to the ligation. Additionally, the small-scale 3C ligation used 1.5 × 10^5^ nuclei in a 1.5 ml reaction volume. Each large-scale 3C was performed once, while the small-scale 3C was performed with three biological replicates.

Relative-enrichment (RE) was assessed with quantitative Real-time polymerase chain reaction (PCR) using FastStart SYBR Green Master reagent (Roche Applied Science, Indianapolis, IN, USA) on a Mastercycler Realplex 2 (Eppendorf AG, Hamburg, Germany), comparing the enrichment of a primer pair from 400 ng of 3C library relative to 40 ng of a randomly ligated library of Bacterial Artificial Chromosome fragments covering the region of interest. See Supplementary Table S2 for the bacterial artificial chromosome (BAC) clones used to create the BAC control library. Each Real-time PCR was done with four independent measures. Primers were designed with Primer 3 (39), tiled across all EcoRI restriction sites from chr9:21,714,180 to chr9:22,347,420, excluding any primers that overlapped with repeat elements as predicted by RepeatMasker (40). See Supplementary Table S1 for primer details.

**Figure 1.**
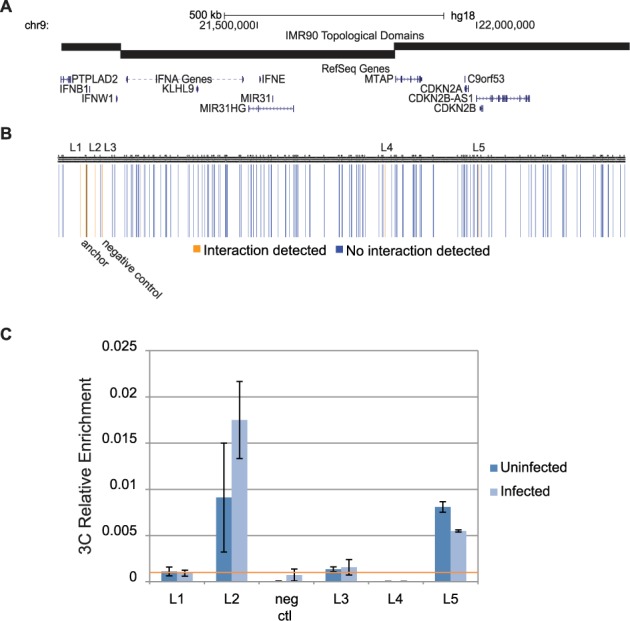
Identification of four sites interacting with the promoter of IFNB1. (**A**) The region of chromosome 9 queried against the promoter of IFNB1 in the 3C assay (chr9: 21 002 063–22 347 415) is displayed against the genes within the locus and the topological domains in the locus (45). (**B**) Results from the single-replicate preliminary 3C study are visualized as vertical bars representing the queried restriction site. Yellow bars represent sites that generated a PCR product in either the uninfected or infected state, while blue bars indicate sites with no PCR product in either condition. The black bar represents the anchor site at the promoter of IFNB1 (chr9:21068221) and is indicated along the bottom of the panel. Sites scoring positively that were >15 kb from the anchor site are labeled long the top of the panel. A site that scored negative and was used as a negative control in the validation experiment is indicated below the bars. (**C**) 3C results were validated with three independent biological replicates in semi-quantitative Real-time PCR. The black horizontal line indicates an RE relevance cutoff point at 0.001. Any sample that exceeded this threshold was considered to interact with the promoter of IFNB1.

Because of the higher repeat density of this locus (40), we could not place intervening primers between IFNB1 and sites L1 and L2, precluding us from using the typical approach for assessing the significance of a chromosomal interaction (41). However, we noted that, in other 3C work, the decay was substantial by 15 kb from the anchor point (15,42–43), and both sites I and IV are >15 kb away from the anchor site (Figure 1A and B). Thus, we compared them to our RE threshold of 0.001.

### Analysis of previously published genomic data

All human genomic analysis was performed on the hg18 build, while mouse analysis was done with the mm9 build. All visualizations were done with the University of California at Santa Cruz (UCSC) Human Genome Browser (44). Data for the IMR90 topological domains were retrieved from previous work (45). Digital DNase and chromatin immunoprecipitation sequencing (ChIP-seq) analysis was performed with the data from the ENCODE project (31), unless otherwise specified. Orthologous regions of the mouse genome were identified with the ‘liftover’ tool in the UCSC Human Genome Browser. An orthologous region to L3 in the mouse genome was further sought using Blastn (46).

The Conserved Elements and Multiz Alignment data was previously published (47–49), while the transcription factor binding sites (TFBSs) were predicted from the TRANSFAC database (50). Human-mouse direct alignment and sequence comparisons were performed with Geneious Alignment Free Gap End Global Alignment tool (Needleman–Wunsch variant (51)) in the program Geneious (Biomatters Ltd., Auckland, New Zealand) using default settings.

### Generation of reporter constructs

All digestion and ligation reactions were performed with enzymes from New England Biolabs. Promoter-testing constructs used enzymes KpnI and XhoI to sub-clone L2 into pGL3-basic, with XhoI and HindIII used to subclone the IFNB1 −110 region into it. Enhancer-testing constructs used SalI and BamHI to sub-clone in the fragment of interest into either pGL3-promoter or pGL3–110. In exception to this, subcloning of 4L2F utilized XhoI only, and was ligated into the compatible SalI site in pGL3-promoter. Please see Supplementary Table S1 for primers associated with each construct, with primers designed by Primer 3 (39).

Mutagenesis was performed according to the guidelines provided in the QuikChange Site-Directed Mutagenesis Kit (Agilent Technologies, Santa Clara, CA, USA), with the following variations. PCR utilized a final dNTP concentration of 2 mM (Roche Applied Science). PCR was performed for 16 cycles. The final DpnI digested product was transformed into electrocompetent TOP10 cells generated by an existing protocol (52). Please see Supplementary Table S1 for mutagenesis primer details.

### Luciferase assays

Luciferase assay conditions followed standard protocols associated the Dual-Glo Luciferase Assay System (Promega, Madison, WI). Luciferase counts were read either on the Perkin-Elmer Victor^3^ V Plate reader, Turner Biosystems Modulus Luminometer 9200–001 or the Turner Biosystems Veritas Microplate Luminometer 9100–002. For any samples with independent replicates of at least nine, the highest and lowest data points were excluded. The sample size listed in the caption accounts for the two removed data points.

### Chromatin immunoprecipitation

ChIP was performed using standard protocols (38) on >1 × 10^6^ cells per immunoprecipitation, with an SMC1A antibody (Bethyl Labs, catalog # A300–055A), a Med1 antibody (Bethyl Labs, catalog # A300–793A), an RNAPII antibody (Millipore, catalog # 05–623) and a phospho-IRF3 antibody (Cell Signaling, catalog # 4947). See Supplementary Table S1 for primer sequences, designed with Primer 3 (39). Real-time PCR was performed as described above (see 3C), using 500 pg of ChIP DNA per reaction. If the starting sample size was ≥5, and the removal of a single data point reduced the standard error by over 20%, that data point was excluded as an ‘outlier.’ In these instances, no more than one data point was removed.

### siRNA treatment

Each siRNA treatment included a final concentration of 40nM siRNA on both uninfected and SeV treated HEK293 cells. We used siGENOME Non-Targeting siRNA #3 (Dharmacon, Lafayette, CO, catalog # D-001210–03–05) as a negative control. We used a pool of two custom-synthesized ds-siRNAs for each eRNA, with each siRNA at equal concentration. RNA extraction and oligodT-primed cDNA synthesis was performed as previously described (38). Please see Supplementary Table S1 for siRNA sequences.

### GRO-seq analysis

Uninfected and SeV-infected IMR90 cells were subjected to GRO-seq, with mapping and alignment to the hg18 human genome performed with bowtie, according to previously published protocols (53).

### BAC recombineering

Recombineering was performed according to previously published protocols (52) using the clone CTD2104N16 (Life Technologies). Oligos used for BAC recombineering are detailed in Supplementary Table S1.

## RESULTS

### Looping identifies candidate *cis*-regulatory elements of IFNB1

Actively engaged *cis*-regulatory elements form chromosomal loops to their target genes (15). We therefore used an unbiased *cis*-loop detection approach to identify candidate regulatory elements associated with IFNB1. We established a two-tiered 3C-based approach to identify candidate regulatory elements that interact with the promoter of the IFNB1 gene. Because IFNB1 expression is induced by virus infection, each of these experiments was performed in both uninfected and SeV-infected IMR90 primary human fibroblast cells.

A preliminary 3C analysis was performed on 5 million cells per condition to identify candidate elements that interact with the IFNB1 promoter, excluding repeat regions, across ∼1.4 mb surrounding the IFNB1 gene on the chromosome 9. We have tested 172 potential interactions with four technical replicate quantitative PCR (qPCR) reactions per site from each of the uninfected and infected 3C libraries, as well as from the BAC control library, for a total of 2064 qPCR reactions. If the 3C-based proximal ligation between the IFNB1 promoter and an interrogated site produced any detectable product in the majority of Real-time qPCR reactions in either the uninfected or infected state, we then selected the site for subsequent validation steps. Our locus-wide 3C analysis identified seven EcoRI restriction fragments as preliminary candidate interacting sites (Figure 1B). Two of these sites are within 15 kb of the anchor site, and were excluded from further analysis because of the background enrichment typically seen for sites near the anchor point (see ‘Materials and Methods’ section for details). The remaining sites were labeled L1–L5. Sites L1–L4 were all intergenic regions, while site L5 included the promoter of the CDKN2B gene and was ∼930 kb away from the IFNB1 promoter. Site L1 was 16.6 kb downstream of IFNB1. Sites L2 and L3 were 19.7 and 36.6 kb upstream of the IFNB1 gene, respectively, with site L4 located 710 kb upstream of IFNB1 (Figure 1A and B).

We pursued these five sites for validation and included an additional site in between sites L2 and L3 as a negative control, as no meaningful interaction was detected between IFNB1 and this restriction fragment (Figure 1C). We set a normalized RE threshold value of 0.001 for validating an interaction. Of the five sites, interactions with L2 and L5 robustly exceeded our RE threshold in both the uninfected and infected states. L1 and L3 only marginally broke this threshold, with L1 only doing so in the uninfected state and L4 was not validated (Figure 1C). Given this, we excluded L4 from further analysis

We then analyzed if the status of any of the chromatin loops were specifically induced by viral infection (Figure 1C). Only the loop to L2 exhibited any notable difference, with a 1.9-fold stronger interaction in the infected state. However, this increase did not pass the nominal statistical significance threshold. Most significantly, all of the validated interaction sites were detectable in both infected and uninfected state, suggesting that the chromatin looping architecture involved in the induction of the IFNB1 gene is most likely established prior to viral infection, consistent with recent observations from HiC analyses (54).

Recent work has shown that the genome is organized into large-scale topological domains, and that most chromosomal interactions occur within a single topological domain (45). Because of this, we mapped the interaction profile of IFNB1 to the topological domains previously identified in IMR90 (Figure 1A and B) (45). Three sites, L1–L3, were all located within the same topological domain as the IFNB1 gene. Only site L5 is located in a different topological domain. These results are consistent with the previous observation that most chromatin loops exist within a single topological domain rather than across two separate topological domains (54).

### Chromatin state and conservation screens candidates for putative long-range enhancers of IFNB1

**Figure 2.**
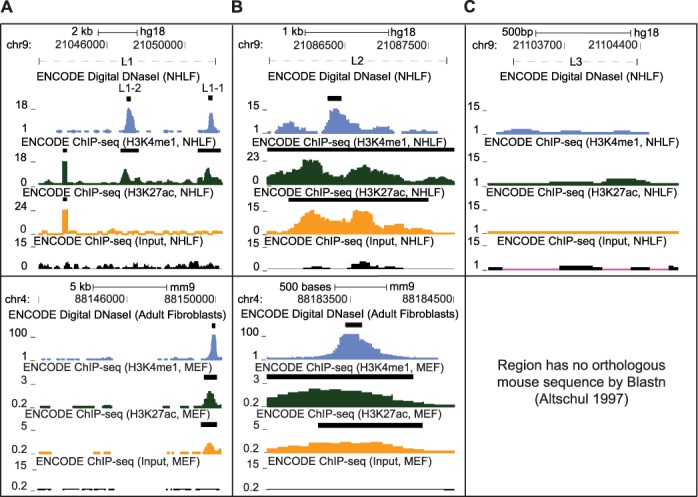
L1 and L2 include sites with chromatin markings of enhancer activity conserved to mouse. (**A**) DNase Hypersensitivity, H3K4me1, H3K27ac and ChIP input enrichment plots and peaks from ENCODE were assessed for L1 both in normal human lung fibroblasts (NHLF, top half), as well as against the chromatin state in the orthologous mouse region in embryonic fibroblast (MEFs) or adult fibroblasts (bottom half) (31,44). (**B**) Chromatin state was assessed for L2 as in (A). (**C**) Chromatin state for L3 was assessed for human fibroblasts as in (A). (A and B) Black bars atop each enrichment plot indicate previously called enrichment peaks.

Excluding the site L5 as a promoter for CKDN2B, we focused on sites L1–L3 as our candidate enhancers. We assessed the levels of H3K4me1 and H3K27ac enrichment, along with DNase I hypersensitivity of these sites to further evaluate these candidate enhancers. Previous work has demonstrated that, upon virus infection, p300 is recruited to sites that are pre-marked as enhancers (55), indicating that the chromatin state in uninfected cells would be informative. We used chromatin immunoprecipitation and DNase I hypersensitivity coupled to high-throughput sequencing ( and Digital DNase, respectively) data generated by the ENCODE consortium for uninfected Normal Human Lung Fibroblast cells (NHLFs), a cell type highly similar to IMR90s (Figure 2, top half). We observed two DNase I hypersensitive sites (DHS) in site L1, and refer to the IFNB1-proximal L1-DHS as L1–1 and the IFNB1-distal L1-DHS as L1–2. Although both of these DHS within L1 display H3K4me1 enrichment, neither displays H3K27ac enrichment (Figure 2A). Fragment L2, on the other hand, included a DHS with both an H3K4me1 and an H3K27ac peak (Figure 2B). Site L3 did not include a DHS, H3K4me1 or H3K27ac enrichment (Figure 2C). We also examined previously published H3K4me1 and H3K27ac enrichment patterns in uninfected IMR90 cells (56), observing the same pattern for all three sites in this cell line. Given the lack of enhancer-associated chromatin characteristics, we ruled out site L3 as a putative enhancer.

To further refine our collection of regulatory elements, we then assessed the conservation of chromatin state of sites L1, L2 and L3 to their mouse orthologs (Figure 2, bottom half). Using the liftover tool in the UCSC Genome Browser (31), we identified orthologous sequences to L1 and L2 in the mouse genome, but not L3. We further tested the sequence for L3 using the *blastn* algorithm to search for similar murine genomic sequences more comprehensively (46), but our effort did not yield significant hits (data not shown). From these results, we conclude that L3 lacks a mouse ortholog.

Based on ChIP-seq and Digital DNase results from the ENCODE Project (31), the orthologous sequences to the L1–1 and L2 also exhibit DNase I hypersensitivity in mouse fibroblasts (Figure 2A and B). Both of these murine sites exhibit enrichment for H3K4me1 and H3K27ac as well (Figure 2A and B). This conservation of chromatin structure provides additional support for functional relevance of the sites L1–1 and L2.

**Figure 3.**
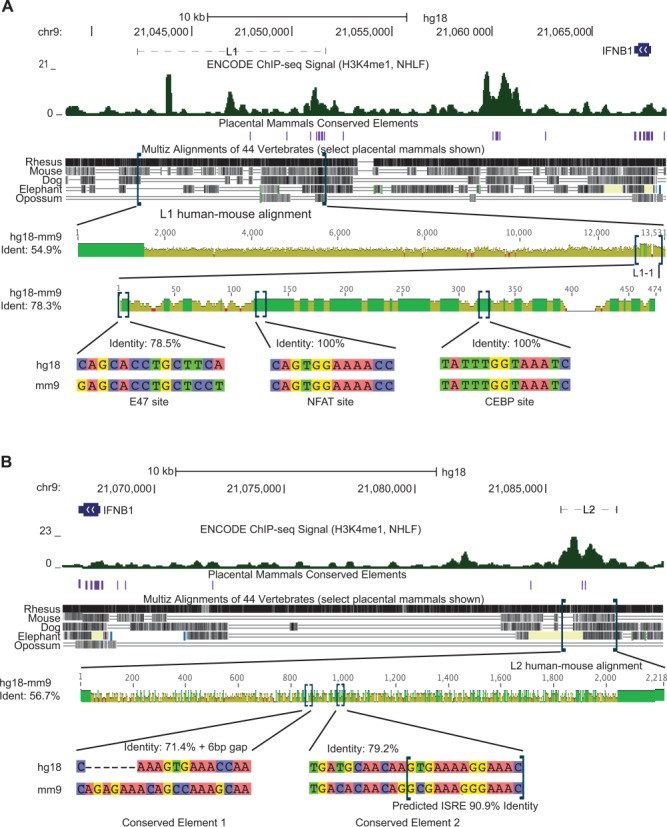
L1–1 and L2 include elements that are highly conserved to mouse at the sequence level. (**A**) Sequence conservation of L1. Top Section: The L1 genomic context was plotted against human DNA elements conserved across placental mammals, as well as against the alignment of this region to five different mammalian species, with H3K4me1 enrichment included for frame of reference to Figure 2. Second Section: L1 was aligned to its mouse ortholog and assessed for nucleotide-level sequence conservation. The height of the bar represents nucleotide identity at the centerpoint of a 5 bp window. Third Section: L1–1 was aligned to its mouse ortholog and assessed for nucleotide-level sequence conservation as in the second section. Bottom Section: Three predicted transcription factor binding sites were assessed for sequence conservation to their orthologous sequences in mouse. (**B**) Sequence Conservation of L2. Top and Middle Sections: Sequence conservation of L2 was assessed as in the top two sections of Figure 3A for L1. Bottom Section: The two conserved elements in L2 were assessed for their sequence conservation to their orthologous sequences in mouse, along with the sequence conservation for the TRANSFAC-predicted ISRE. (A and B) Blue brackets indicate the region that will be expanded in the following section.

To assess L1 in more detail, we examined its sequence conservation at nucleotide resolution (Figure 3A). The Multiz alignment of placental mammalian genomes (47) indicated that the L1–1-associated H3K4me1 peak (31) is one of only three regions with the syntenic sequence that aligns across all five species examined. L1–1 also included four sequence elements that were conserved across placental mammals (48). We then performed a sequence alignment of the human and mouse orthologous regions of L1 with the Geneious Aligner (free gap end variant of Needleman–Wunsch (51)). Alignment of the human L1 fragment with its mouse ortholog revealed 54.9% sequence identity. L1–1, however, exhibited 78.3% sequence identity. A previously established catalog of predicted TFBS (50) indicates three distinct conserved TFBS within the L1–1. The predicted E47, NFAT and CEBP sites were highly conserved between human and mouse sequences. Both NFAT and C/EBPβ play key roles in innate immune activity (57,58), and E47 has well-documented roles in B cell development (59). However, we found no report of any of these factors directly regulating Type-I interferon expression, and no predicted binding sites for either NFκB or the IRF-family of transcription factors, both of which are known to regulate IFNB1 transcription (28).

To assess L2 in more detail, we performed a similar sequence analysis as with L1 (Figure 3B). L2 contains two conserved elements (48) and exhibits a broad region of synteny for four of the five species displayed (47). The alignment to mouse revealed 56.7% sequence identity. We isolated the individual conserved elements, finding them to exhibit >70% sequence identity between human and mouse, though one of the elements had a 6 bp insertion in mouse. Finally, and most significantly, one of the conserved elements within L2 included a predicted Interferon Stimulated Response Element (ISRE) (50). This element, a known component of the antiviral response (30), shared 10 out of 11 identical bases between human and mouse.

### L1–1 and L2 recruit relevant transcription factors in a virus-inducible fashion

**Figure 4.**
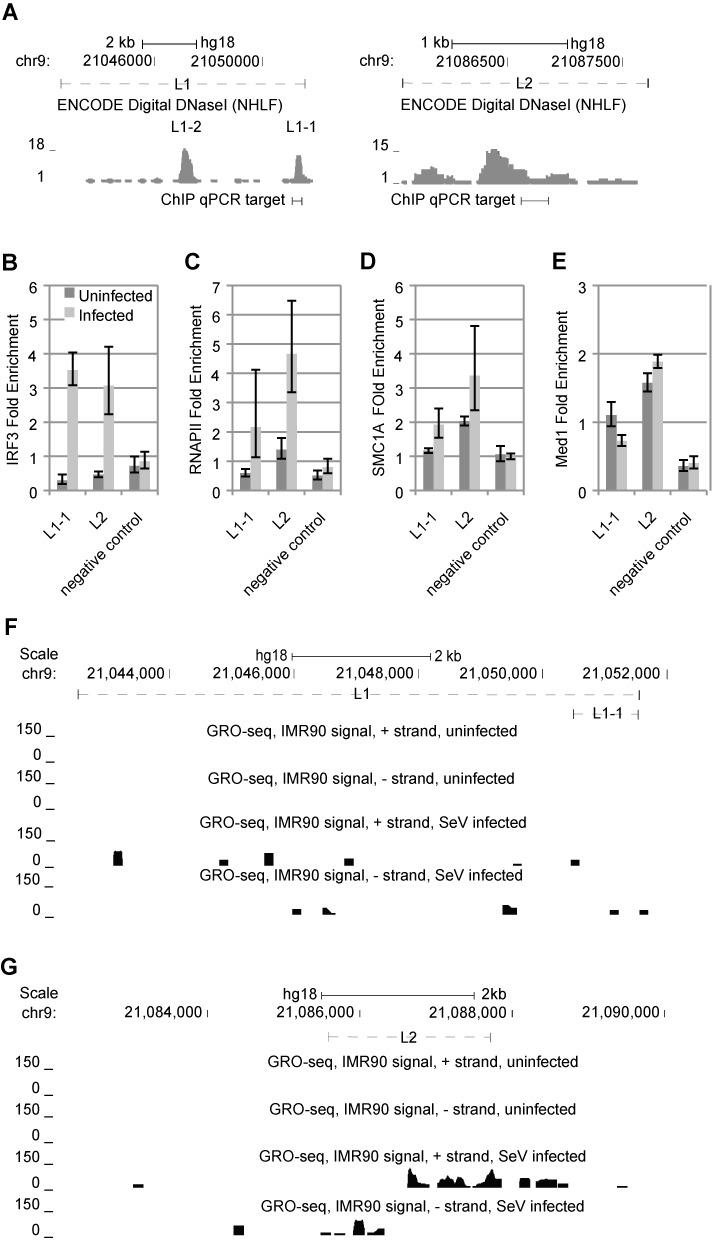
Virus-inducible transcription factor recruitment and eRNA production at L1 and L2. (**A**) ChIP-PCR primers were designed to detect transcription factor enrichment within the DHS of L1–1 (left) and L2 (right). (**B**–**E**) Enrichment of TF binding binding enrichment over input chromatin at L1, L2 and a negative control site were assessed in uninfected (dark gray) and virus-infected (light gray) IMR90 cells for pIRF3 (B), RNAPII (C), SMC1A (D) and Med1 (E). Each column represents at least three independent experiments. Error bars represent standard error. (**F** and **G**) GRO-seq was performed both in uninfected as well as infected IMR90 cells. The EcoRI sites represent the outer bounds of the restriction fragment that defines L1 (**F**) and L2 (**G**). GRO-seq output was visualized with normalized RPM (RNA tags per million sequenced) multiplied by 100 plotted against the L2 region at single-nucleotide resolution. Top two tracks show the GRO-seq data from uninfected cells. The next two tracks show that for the SeV-treated state.

To further assess the *in vivo* functional relevance of L1 and L2, we analyzed the recruitment of the phosphorylated version of the key IFNB1-regulating transcription factor IRF3 (pIRF3) (60), RNAPII (61), Cohesin component SMC1A (16) and Mediator component Med1 (62), to L1–1 and L2 (Figure 4A) by ChIP. ChIP analysis determined that both pIRF3 and RNAPII exhibited no enrichment relative to input chromatin in the uninfected state at either L1–1 or L2, but found pIRF3 consistently recruited to both sites in the infected state (Figure 4B and C), and RNAPII only recruited to L2 in a consistent manner. Interestingly SMC1A exhibited constitutive enrichment at L2, but not L1–1, and exhibited modest virus-induction of recruitment to both of these sites (Figure 4D). Med1 also exhibited modest baseline enrichment at site L2, but exhibited no induction of recruitment in response to viral infection (Figure 4E). In addition, previous work has observed IRF3, NFκB and RNAPII enriched at L2 in SeV-treated Namalwa Burkitt's Lymphoma cells (63). Interestingly, they did not observe these factors enriched at L1. The virus-inducible recruitment of these transcription factors to L2 further suggests that it may be functionally relevant to the regulation of IFNB1.

### L2, but not L1, produces eRNAs in a virus-inducible fashion

Given the discovery of eRNAs as an indicator of enhancer activity (19,20), we wanted to determine if either L1 or L2 produced eRNAs in a virus-inducible fashion. We evaluated genome-wide nascent transcription using GRO-seq analysis of both the uninfected and infected IMR90 cells. We recovered 25,761,353 and 25,432,390 tags, which resulted in 12 545 400 and 16 746 169 mapped reads to the hg18 genome in the uninfected and infected states, respectively. We visualized the GRO-seq mapped reads on the UCSC human genome browser (Figure 4F and G, (44)).

eRNAs exhibit a characteristic transcriptional signature of bi-directional transcription emanating outward from the enhancer element (18). In the uninfected state, both L1 (Figure 4E) and L2 (Figure 4F) were transcriptionally silent, indicating no eRNA production in the uninfected state. In the virus-infected state, L1 exhibited low levels of sparse transcription distributed across the fragment (Figure 4E). This is not consistent with the known pattern of eRNA transcription (18). From L2, however we observed robust, bi-directional transcription emanating outward from the of the L2 element, proceeding <3 kb in either direction (Figure 4F), consistent with previously characterized eRNAs (18,19).

We looked for evidence of other transcriptional units at L2 that would be consistent with mRNAs. However, we found no annotated genes near L2 from either the Refseq (44,64) or Ensembl (44,65) annotations. Furthermore, the aggregated ‘Human EST’ track from UCSC Genome Browser revealed no expressed sequence tags at this site (65). We also found no evidence of predicted polyadenylation signals in or near L2, with the nearest polyadenylation sites belonging to the neighboring genes IFNB1 and IFNW1 (44,66–67). Thus, this transcriptional unit has not been annotated before, and based on available evidence, this transcriptional unit is unlikely to be an mRNA.

### L1–1 has neither promoter nor enhancer activity

**Figure 5.**
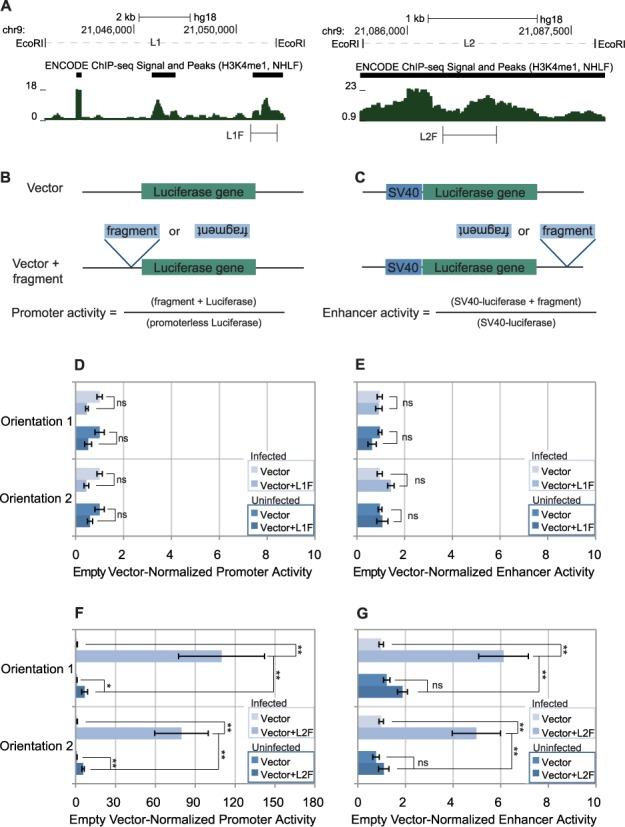
L1 is inert, but L2 includes both promoter and enhancer activity in a virus-inducible fashion. (**A**) The location of the L1F and L2F fragments amplified for the luciferase assays are show at the bottom of the panel, plotted against H3K4me1 enrichment in (Figure 2) for reference. L1 is shown on the left, L2 is shown on the right. (**B**) For luciferase promoter activity assays, the fragment of interest is placed immediately upstream of a promoterless luciferase gene (pGL3-basic). To calculate promoter activity, the luciferase activity of the fragment-driven reporter is divided by the activity of the promoter-less reporter. (**C**) For Luciferase enhancer activity assays, the fragment of interest is immediately downstream of an SV40-driven luciferase reporter (pGL3-promoter). To calculate enhancer activity, the luciferase activity of the fragment-enhancer SV40-driven reporter is divided by the activity of the SV40-driven reporter. (**D**) Promoter activity of L1F in both orientations, before and after virus infection. All constructs were analyzed in at least three independent experiments (n ≥ 3). (**E**) Promoter activity of L1F in both orientations, before and after virus infection. All constructs were analyzed in at least seven independent experiments (n ≥ 7). (**F** and **G**) Luciferase promoter and enhancers assays were performed as in (D and E) respectively, but with L2F instead of L1F. All constructs were analyzed in at least seven independent experiments (n ≥ 7). (D–G) Error bars indicate Standard Error. We used unpaired t-*t*ests to assess statistical significance. A single asterisk, ‘*’, indicates *P* < 0.05. A double asterisk, ‘**’, indicates *P* < 0.01. The letters ‘ns’ mean ‘not significant and indicate *P* > 0.05.

We tested a 563-bp fragment of L1–1 that contains conserved DHS, which we call L1F, for promoter activity by reporter assays (Figure 5A and B). In the both uninfected and virus-infected state, we found that luciferase activity driven by L1F was comparable to luciferase expression detected in the absence of the element (Figure 5D). We subsequently tested L1F in an enhancer assay by cloning it downstream of the SV40 promoter-driven luciferase gene. We observed that L1F had no impact on luciferase activity in this assay in either the uninfected or virus-infected state (Figure 5E). From these results, we concluded that L1F lacks any relevant promoter or enhancer activity in either the uninfected or virus infected state. Between these results and the lack of eRNA production (Figure 4E), we did not pursue L1 for further analysis.

### L2 exhibits both virus-inducible enhancer and promoter activity

We placed a 543-bp fragment of L2 centered on the DHS, which we called L2F, downstream of the SV40 promoter-driven luciferase gene in the reporter construct to assess its enhancer activity (Figure 5A and C), again testing both orientations (Figure 5G). In both orientations, L2F had no significant impact on luciferase expression. However, upon virus infection, L2F displayed significant enhancer activity (*P* = 2.4 × 10^−6^ and 6.4 × 10^−4^), representing 3.2-fold and 4.5-fold virus inducibility (*P* = 0.0018 and 9.9 × 10^−4^) in orientations 1 and 2, respectively. The difference in activity between orientations was not statistically significant. These results demonstrate that L2 contains orientation-independent virus-inducible enhancer activity, as expected from an enhancer associated with the IFNB1 gene.

Based on our detection of eRNAs by GRO-seq, we were curious about whether or not L2 exhibited any promoter activity. We tested this by placing L2F upstream of a promoter-less luciferase gene. (Figure 5F). In both orientations, L2F exhibited a surprising level of promoter activity that was weak but significant (*P* = 0.0021 and 1.2 × 10^−5^ in orientations 1 and 2, respectively). Upon virus infection, L2F drove dramatically higher promoter activity in both orientations 1 and 2 (*P* = 0.0035 and 2.6 × 10^−4^ respectively). These represented virus-inducibility of 16-fold (*P* = 0.0052) for orientation 1 and 14-fold (*P* = 0.0032) for orientation 2. We found no meaningful difference in activity between orientations. These results demonstrate that L2 contains orientation-independent, bidirectional basal promoter activity that is robustly virus-inducible.

### L2 transcriptional activity is dependent on the ISRE nucleotide sequence

**Figure 6.**
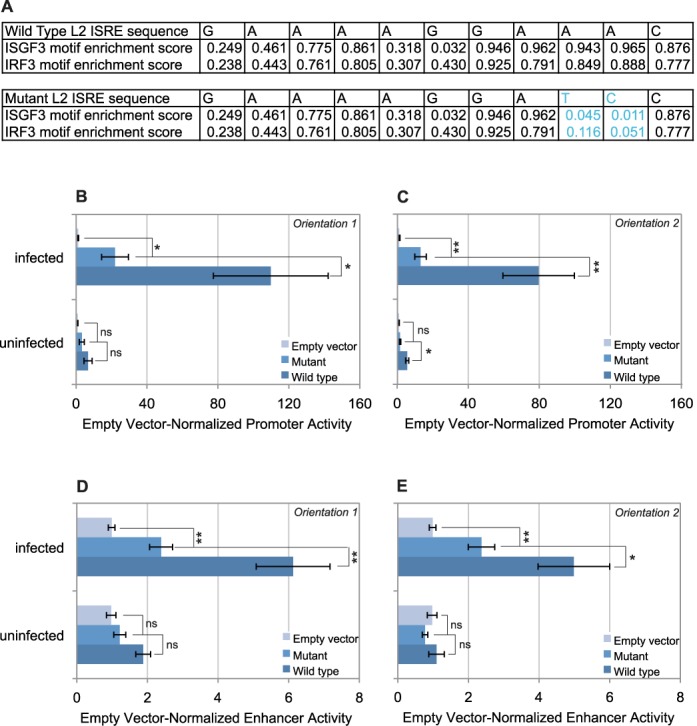
A 2-bp ISRE mutation impairs, but does not abolish, L2F's virus-induced transcriptional activities. (**A**) Top half: The specific nucleotides of the L2 ISRE sequence were assessed for their enrichment score in the primary motifs for ISGF3 and IRF3 from the Uniprobe database (68). Bottom half: The specific nucleotides of the mutated L2 ISRE sequence were assessed for their enrichment score in the primary motifs for ISGF3 and IRF3 from the Uniprobe database (68). (**B**) ISRE mutation effect on the promoter activity was determined for Orientation 1. All constructs were analyzed in 10 independent experiments (*n* = 10). (**C**) ISRE mutation effect on the promoter activity was determined for Orientation 2. All constructs were analyzed in seven independent experiments (*n* = 7). (**D**) The impact of the ISRE mutation on enhancer activity was determined for Orientation 1. All constructs were analyzed in at least seven independent experiments (*n* ≥ 7) **E.** Impact of ISRE mutation on enhancer activity was determined for Orientation 2. All constructs were analyzed in 13 independent experiments (*n* = 13). (B–E) Error bars indicate Standard Error. We used unpaired t-*t*ests to assess statistical significance. A single asterisk, ‘*’, indicates *P* < 0.05. A double asterisk, ‘**’, indicates *P* < 0.01. The letters ‘ns’ mean ‘not significant and indicate *P* > 0.05.

Our sequence analysis had revealed that L2 contains a conserved ISRE (Figure 3). In the Type-I interferon response, ISRE sequences are typically bound by ISGF3 (30), but there is no evidence that ISGF3 directly regulates IFNB1. However, IRF3 plays a key role in the activation of IFNB1, binding to the IFNB1 proximal enhancer (26) and has a very similar binding specificity to ISGF3 (68). Moreover, we observed inducible recruitment of IRF3 to L2 (Figure 4B). Because of this, we hypothesized that the integrity of the ISRE was necessary for the activities of L2. Therefore, we subjected the ISRE to site-directed mutagenesis and tested the mutant in our luciferase assays. However, it was not yet certain if the element was regulated by IRF3 or ISGF3. To mutate L2, we therefore mapped the L2 ISRE sequence against their nucleotide-level enrichment scores for previously defined binding motifs of both ISGF3 and IRF3 (68) (Figure 6A). Both motifs displayed very stringent requirements for the three sequential adenosine nucleotides at the 3′ end. We made a minor perturbation, mutating the ‘AA’ to ‘TC’ (Figure 6A), to each L2 construct used in Figure 5.

Each mutated L2 construct was compared against the wild-type and empty-vector versions of the construct, first assessing promoter activity. In orientations 1 and 2 (Figure 6B and C), we observed that mutant L2F was indistinguishable from the wild-type in the uninfected state. Upon virus infection, the mutant L2F exhibited moderate promoter activity, representing a marked reduction to 15–20% of wild-type L2F activity levels (*P* = 0.016 and *P* = 0.0067). There was no meaningful difference between the activities of the different orientations of mutant L2F. Together, these results suggest that the integrity of ISRE significantly contributes to L2′s promoter activity.

To assess the impact of this mutation on L2′s enhancer activity, we performed a similar set of luciferase assays in enhancer position. In the uninfected state, mutant L2F had no significant enhancer activity in either orientation (Figure 6D and E), much like wild-type L2F. In the virus-infected state, however, this construct exhibited <50% of the enhancer activity of wild-type L2F in both orientations 1 and 2 (*P* = 0.0051 and *P* = 0.023, respectively). Taken together, these results demonstrate that the ISRE is a major contributor to the transcriptional activities of L2. The involvement of the ISRE sequence is consistent with our finding that IRF3 is inducibly recruited to L2 (Figure 4), and consistent with our hypothesis that L2 regulates IFNB1 expression.

We also assessed whether or not the residual activities of mutant L2F relative to baseline in the infected state were statistically relevant. Indeed, the infected state promoter activities (Figure 6B and C) in orientations 1 and 2 were both significantly higher than baseline activity (*P* = 0.012 and 4.1 × 10^−4^, respectively). Likewise in the infected state, the retained enhancer (Figure 6D and E) activities of orientations 1 and 2 were both statistically significant (*P* = 5.3 × 10^−5^ and 0.0016, respectively). This indicated that, while the 2 bp mutation had a significant impact on overall virus-inducible promoter and enhancer activities of L2, it was insufficient to completely ablate either activity from this element. Thus, mutant L2F retains low but significant residual activities that are independent of this mutation.

Knowing that L2's activity was dependent on the ISRE, it remained ambiguous whether or not L2 was regulated by IRF3 or ISGF3. Given that virus infection activates IFNβ expression that in turn activates ISGF3 (30), we hypothesized that if the element's activities were driven by ISGF3, it should be responsive to both SeV infection as well as IFNβ treatment. However, if L2′ activities are IRF3-driven, it should respond only to SeV treatment. Given that IFNB1 is regulated by IRF3 (26), and that we observed IRF3 inducibly recruited to L2 (Figure 4), we hypothesized that this element would be driven by IRF3 and therefore would be virus-responsive, but not IFNβ-responsive. Because of this, we tested if the wild-type L2 constructs responded to IFNβ treatment. We found that IFNβ treatment had no impact on L2F's promoter or enhancer activity (Supplementary Figure S1), while the same IFNβ treatment robustly activated endogenous ISGs (data not shown). This implies that L2′s activities are not regulated by ISGF3, and, in combination with our ChIP data, suggests that the activities of L2 may be driven by IRF3.

### L2 is capable of acting on the IFNB1 promoter and proximal enhancer

**Figure 7.**
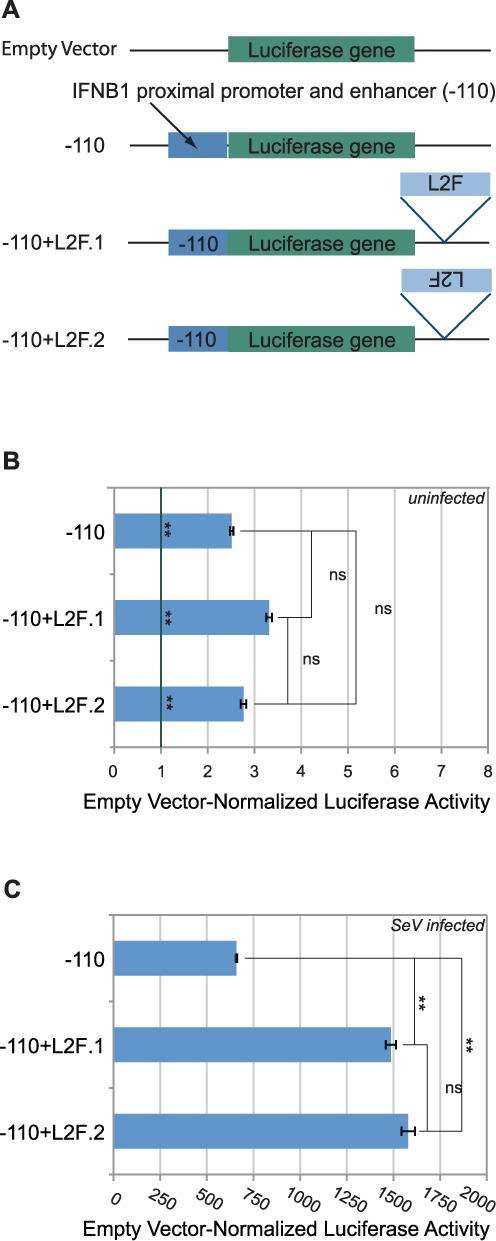
L2F enhances the activity of the previously known IFNB1 proximal regulatory elements. (**A**) Four constructs were used for the luciferase assay. The endogenous IFNB1 promoter and proximal enhancer together (−110) were placed upstream of the Luciferase reporter gene. L2F was inserted downstream of the reporter gene in one of two orientations. (**B**) The Luciferase activity of each test construct was examined in uninfected cells. The vertical line across all three bars represents baseline activity. Significance markings adjacent to this line indicates that the construct indicate differences between the marked bar and baseline activity. Each construct was tested in at least three independent experiments (*n* ≥ 3). (**C**) The Luciferase activity of each test construct was examined in SeV-infected cells. Each construct was tested in at least three independent experiments (*n* ≥ 3). (B and C) Error bars indicate Standard Error. We used unpaired t-*t*ests to assess statistical significance. A single asterisk, ‘*’, indicates *P* < 0.05. A double asterisk, ‘**’, indicates *P* < 0.01. The letters ‘ns’ mean ‘not significant and indicate *P* > 0.05.

If L2 regulates IFNB1 *in vivo*, then L2 and the previously characterized IFNB1 proximal regulatory elements (26), the −110 element, should be able to functionally interact in a reporter context. To explicitly test this prediction, we cloned the −110 element immediately upstream of a promoter-less luciferase reporter gene. We compared the expression of this construct to that of L2F inserted downstream of the −110-driven luciferase (Figure 7A). In the uninfected state (Figure 7B), −110 element alone conferred a promoter activity of 2.5. The addition of L2F had no meaningful impact in either orientation in the uninfected state.

The virus-infected state revealed that L2F could act as an enhancer for the −110 element (Figure 7C). The −110 element alone drove robustly virus-inducible promoter activity (*P* = 9 × 10^−11^). The inclusion of L2F further increased luciferase activity by more than 2-fold in both orientations (*P* = 5.2 × 10^−6^ and *P* = 9.4 × 10^−6^ for orientation 1 and 2, respectively). Thus, L2 is capable of enhancing the activity of the −110 element, as expected of an enhancer that would regulate IFNB1 transcription.

### L2 retains its reconstituted activities in the absence of eRNA sequences

**Figure 8.**
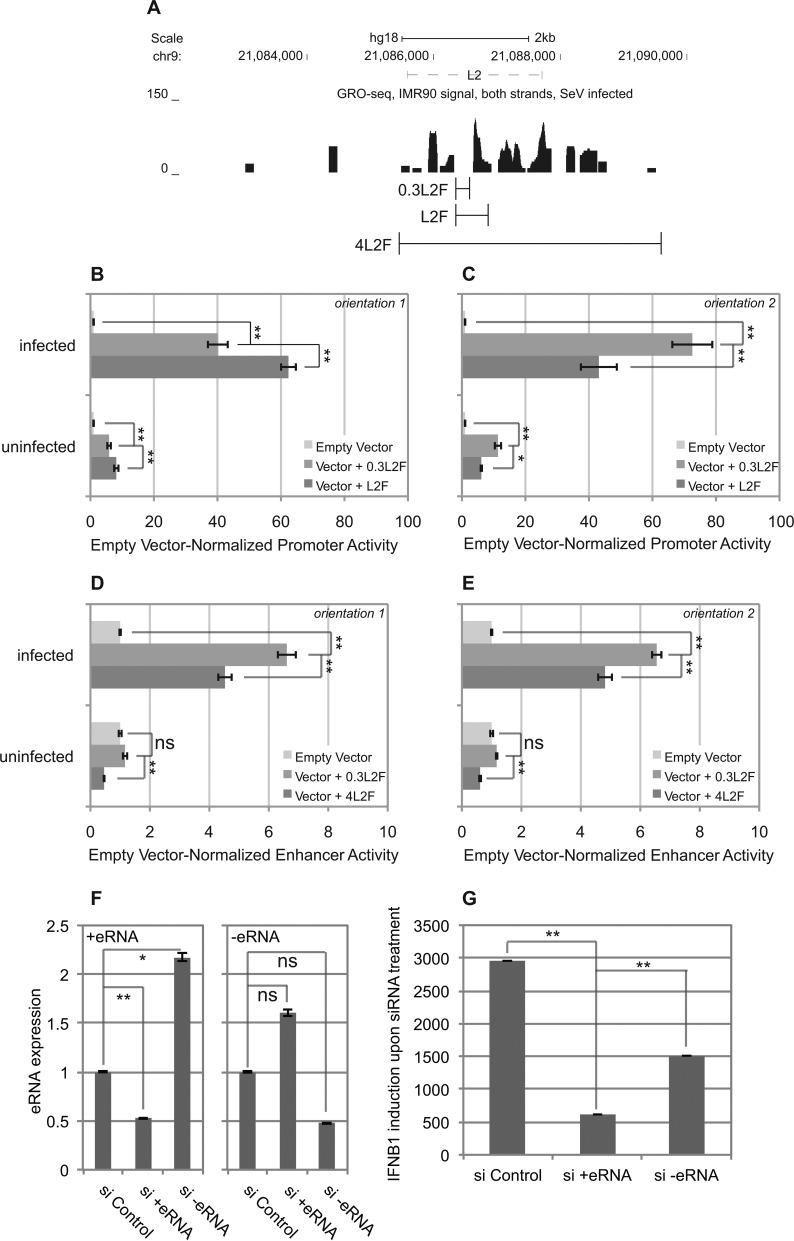
The L2 fragment lacking eRNA sequences retains transcriptional activities, even though the eRNA is necessary for endogenous IFNB1 expression. (**A**) The genomic fragments used for the L2 fragment (L2F) and the eRNA-excluding L2 fragment (0.3L2F) and the larger eRNA-inclusive fragment (4L2F) are shown against the GRO-seq reads at L2 for reference (from Figure 4). For the GRO-seq data, data from both strands are displayed in a single track. (**B**) The impact of the exclusion of remaining eRNA sequence was assessed on L2 promoter activity in Orientation 1. (**C**) The impact of the exclusion of remaining eRNA sequence was assessed on L2 promoter activity in Orientation 2. (**D**) The impact of the presence or absence of eRNA sequence was assessed on L2 enhancer activity in Orientation 1. (**E**) The impact of the presence or absence of eRNA sequence was assessed on L2 enhancer activity in Orientation 2. (B–E) All constructs were analyzed in five independent experiments (*n* = 5). Error bars indicate Standard Error. (**F**) The effectiveness of both siRNA pools on knockdown of SeV-induced eRNA expression levels from each strand was assessed. (**G**) The effectiveness of treatment with each siRNA pool on IFNB1 induction in response to SeV was assessed. (F and G) Expression levels were normalized to GAPDH. Each treatment was tested in three independent experiments (*n* = 3). Error bars indicate Standard Deviation. (B–G) We used unpaired t-*t*ests to assess statistical significance. A single asterisk, ‘*’, indicates *P* < 0.05. A double asterisk, ‘**’, indicates *P* < 0.01. The letters ‘ns’ mean ‘not significant and indicate *P* > 0.05.

We had initially defined L2F based on the location of the DHS in the segment. However, when we mapped L2F GRO-seq transcription unit (Figure 8A), we noticed that only the IFNB1-distal end of L2 contained some sequence that overlapped with the GRO-seq defined eRNA sequences. Since we showed that L2 promoter and enhancer activities were orientation-independent (Figure 5), we speculated that L2′s observed transcriptional activities in luciferase reporter assays might not require the inclusion of adjacent eRNA sequences. To test this hypothesis explicitly, we generated a shorter 317 bp L2 fragment (0.3L2F) completely lacking the eRNA-associated sequences from both ends, as well as a 4160 bp fragment (4L2F) that included the both eRNA-transcribing units (Figure 8A).

To test the necessity of the eRNA sequences to promoter activity, we compared 0.3L2F to L2F. We found that promoter activity of 0.3L2F was robust in the uninfected state for both orientations 1 and 2 (Figure 8B and C, *P* = 2.6 × 10^−5^ and 5.7 × 10^−6^ respectively). While these activities exhibited statistically significant differences to the basal promoter activities of L2F in both orientations 1 and 2 (*P* = 0.033 and 7.7 × 10^−4^, respectively), the magnitude of these differences were weak (0.72-fold and 1.86-fold differences, respectively). In the virus-infected state, 0.3L2F exhibited robustly induced promoter activity for both orientations 1 and 2 (*P* = 1.5 × 10^−6^ and 3.2 × 10^−6^, respectively). In orientation 1, the virus-induced activity of 0.3L2F was lower than that of L2F in a statistically significant manner, but the magnitude of difference was small (*P* = 4.4 × 10–4, 0.64-fold difference). Conversely, the virus-induced enhancer activity of 0.3L2F was higher than that of L2F in a statistically significant manner (*P* = 0.0084). However, it was also a weak difference (1.68-fold). These data show that L2F retains its promoter activity in the absence of its associated eRNA sequences.

We then tested whether the presence or absence of associated eRNA sequences impacted the overall enhancer activity of this fragment, using 0.3L2F and 4L2F. No enhancer activity was detected for 0.3L2F in either orientation in the uninfected state (Figure 8D and E). Interestingly, the inclusion of 4L2F significantly reduced luciferase expression in the uninfected state (*P* = 4.3 × 10^−6^, 64% reduction), in orientation 1 (Figure 8D). It also appeared to reduce expression in orientation 2, but the magnitude impact was very weak (*P* = 1.0 × 10^−7^, 39% reduction, Figure 8E). In the virus-infected state, 0.3L2F did confer enhancer activities in both orientations 1 and 2 (*P* = 4.7 × 10^−8^ and 2.5 × 10^−10^, respectively). These activities were higher than those of 4L2F (*P* = 2.6 × 10^−4^ and 1.1 × 10^−4^), but only marginally (1.45-fold and 1.36-fold difference, respectively). Taken together, we concluded that sL2F retains functional virus-inducible promoter and enhancer activities independent of the inclusion of the eRNA sequences as part of the element in our reporter assays.

### L2-produced eRNAs are necessary for proper IFNB1 expression

Our luciferase assay data suggested the possibility that L2′s enhancer activity might be independent of the eRNAs produced by that element. To test this hypothesis directly, we designed a pool of siRNAs to the eRNAs produced off of each strand, si+eRNA and si−eRNA. We found that each siRNA reduced the expression of its target eRNA by 50% (Figure 8F), which was statistically significant for the si+eRNA (*P* = 0.0048), but did not reach the nominal significance for si−eRNA (*P* = 0.088). In addition, si-eRNA increased the expression the +eRNA by 100% (*P* = 0.016), while si+eRNA appeared to increase the expression of the –eRNA by a 50% factor that did not reach statistical significance (*P* = 0.052). Even though the impacts on the −eRNA did not quite reach significance, we noted that siRNAs against eRNA expression has historically been relatively weak (22–24). Because of this, we then assessed the impact of each siRNA pool on the virus-inducibility of IFNB1 expression (Figure 8G). We found that knockdown of −eRNA reduced IFNB1 induction by 49% (*P* = 0.024), while knockdown of +eRNA reduced IFNB1 induction by 79% (*P* = 0.0025). From this, we concluded that the eRNAs produced from L2 are necessary for the appropriate virus-inducible expression of IFNB1 in its endogenous genomic and chromosomal contexts.

### 0.3L2F contributes to the expression of IFNB1

**Figure 9.**
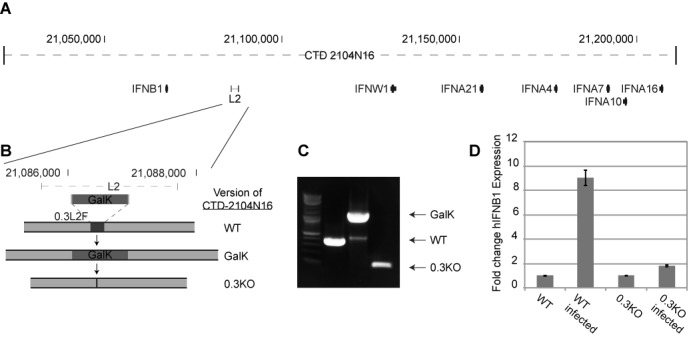
The 0.3L2 fragment is necessary for proper IFNB1 expression. (**A**) The BAC CTD-2104N16 is mapped against the human genome. (**B**) The recombineering process for the deletion of 0.3L2F out of CTD-2104N16 is illustrated. (**C**) Recomineering success was assessed by PCR performed for 0.3L2F on wild-type, GalK-inserted and 0.3KO deleted BAC clones. (**D**) The impact of the 0.3L2F deletion on IFNB1 inducibility was assessed by transient transfection into NIH3T3 cells with or without SeV treatment. Each bar represents two independent experiments (*n* = 2). Error bars indicate one standard deviation.

Having defined 0.3L2F as a minimal unit sufficient to contain virus-induced enhancer activity (Figure 8), we wanted to test if 0.3L2F was necessary for the appropriate virus-inducibility of IFNB1. We used standard BAC recombineering approaches to delete 0.3L2F from the human BAC CTD2104N16, which represents ∼190 kb of chromosome 9 and includes both IFNB1 and the L2 element (Figure 9A and B). PCR from the region adjacent to 0.3L2F indicated that the deletion had been made (Figure 9C). The wild-type and 0.3KO BACs were transiently transfected into NIH3T3 cells, allowing for qPCR-based discrimination between the endogenous mouse IFNB1 (mIFNB1) and the human IFNB1 transgene (hIFNB1). After transfection, we assessed IFNB1 expression in response to SeV treatment, normalized to the degree of induction observed for the endogenous mIFNB1 to control for SeV infection. We noticed a modest elevation of basal expression of hIFNB1 in the uninfected state in 0.3KO BAC when compared to the WT BAC (data not shown). However, the transiently transfected WT BAC exhibited >9-fold induction of hIFNB1 while the 0.3KO BAC exhibited <2-fold induction (Figure 9D). We conclude that 0.3L2F is necessary for the proper expression of IFNB1 in this larger genomic DNA context.

## DISCUSSION

### L2 is a novel enhancer that regulates IFNB1 expression in response to virus infection

We have presented multiple lines of evidence demonstrating that L2 is associated with, and regulates, IFNB1. First, we observed spatial proximity with the IFNB1 promoter (Figure 1). Second, we observed that L2 recruits pIRF3, a key IFNB1-regulating TF (32), in a virus-inducible manner both in IMR90 cells (Figure 4) as well as other cell types (63), while ruling out IFNβ-stimulated signaling as a driver of L2′s activity (Supplementary Figure S1). Third, we observed virus-inducible production of eRNAs from L2 (Figure 4). Fourth, we demonstrated that L2 exhibits virus-inducible enhancer activity that is dependent on the IRF3 binding motif (Figure 6). Fifth, we showed that L2 can cooperate with the IFNB1-proximal promoter and enhancer activity in a reporter context to achieve higher induction levels (Figure 7). Sixth, we have shown that the expression of eRNAs from L2 is necessary for appropriate virus-inducible IFNB1 expression (Figure 8). Finally, we have shown that, in a transient BAC transfection assay, the 0.3L2F element is necessary for proper IFNB1 inducibility (Figure 9). The discovery of this enhancer presents new opportunities to dissect the mechanisms of enhancer action in a biologically relevant context, leveraging what is already known about the mechanisms of classical enhanceosome activity at IFNB1.

### L2′s promoter and enhancer activities are intimately linked

The typical convention to test putative enhancers placed immediately upstream of heterologous promoters (14,69) is based on a general presumption that enhancers exhibit only enhancer activity and no promoter activity. Though the first characterized enhancer, the SV40 enhancer, exhibited very modest transcriptional activity in the absence of a promoter (70), we found no previous record of enhancers demonstrating robust, intrinsic bi-directional promoter activity. Yet, L2 exhibits robust virus-stimulated promoter activity (Figure 5). Moreover, unlike typical promoters (71), it exhibited comparable activity in both orientations. We found it very interesting that both promoter and enhancer activity were hindered by our 2 bp mutation within the ISRE (Figure 6), regardless of orientation. This suggests that both activities might be regulated by IRF3, which we observed binding to this site *in vivo* (Figure 4). That this TFBS is integral to both activities argues that the promoter and enhancer activities are intimately linked, most likely at the molecular level.

### Relationship of eRNAs to enhancer activity is more complex than currently appreciated

We noted that the bi-directional promoter activity of L2 (Figure 5) represents the first evidence of a transcriptional activity that explains the previously observed bi-directional production of eRNAs (18). Indeed, we observed that the eRNAs were biologically relevant *in vivo* (Figure 8). Yet, our data also show that L2′s enhancer activity is reconstituted in luciferase assays independently of the inclusion of the endogenous eRNA sequences *in cis* (Figure 8). It is clear that the production of eRNAs by the enhancer *in cis* is not necessary for enhancer activity as measurable in reporter assays; however, the eRNAs are necessary for high level virus-induced expression of the endogenous IFNB1 gene. Recent work has shown that eRNAs influence chromosomal looping (22)—an activity that might not be relevant in a plasmid-based reporter context. These results emphasize the additional limitations of reporter assays, mainly that eRNA activities are not fully recapitulated in the reporter systems. Additional study could explore if the L2 eRNAs act *in trans*, and to further dissect L2 for various elements that are responsible for eRNA-dependent and eRNA-independent activities in regulating the IFNB1 gene.

It is worth noting that the knockdown of an eRNA in one direction appeared to increase the expression of its opposite-strand counterpart (Figure 8). It may be that the opposite strand eRNA is increased to compensate for the loss of eRNA, though it is not clear by what mechanism this could be occurring. Despite this, knockdown of either eRNA still reduces IFNB1 expression, suggesting that neither eRNA alone is sufficient to contribute the complete eRNA-mediated activities of L2.

### L2 acts within the context of a pre-established and looped chromatin state

Previous work has identified that lipopolysaccharide (LPS) treatment of macrophages, which stimulates Type I interferon production, leads to p300 recruitment to sites that are already premarked for enhancer activity at the chromatin level (55). ENCODE's chromatin data at this locus are consistent with this observation (Figure 2, (31)). In addition, our ChIP data showing the constitutive binding of SMC1A and Med1 and the virus-induced recruitment of IRF3 and RNAPII to the L2 element (Figure 4), along with virus-inducible eRNA transcription from L2 (Figure 4), are the first evidence to suggest that a similar regulatory architecture may govern the antiviral response in non-immune cells as well. More interestingly, we found that, like the associated histone modifications, the chromatin loop connecting L2 to IFNB1 is pre-established (Figure 1). This argues that the chromosomal conformation for the antiviral response, like the TNFα response (54), may be pre-established as well.

These results imply that the cell makes significant steps to establish a transcriptionally quiescent but permissive chromatin state and conformation prior to the immune response, regardless of whether or not it is an immune cell. It seems clear that this chromatin state in immune cells is established by the transcription factor PU.1 (55), and it would be important to investigate what establishes this chromatin state and conformation in non-immune cells. In addition, we find the pre-infection repression of reporter activity by 4L2F (Figure 8) to be interesting. Other groups have found evidence of additional repressive elements outside of the core transcriptional unit (72). It may be worth exploring what repressive units may exist in the flanking sequences of 0.3L2F. Finally, we found it notable that the histone profile of L2 we observed in ENCODE's NHLF data is shared across cell types, including H1 hESCs (31,44,56), suggesting that this chromatin state at L2 is established extremely early in the developmental process.

## ACCESSION NUMBERS

The GRO-seq data have been submitted to the ArrayExpress archive. The accession number for the data is E-MTAB-2233.

## SUPPLEMENTARY DATA

Supplementary Data are available at NAR Online.

SUPPLEMENTARY DATA
