# Supplementary material for: A novel virus-inducible enhancer of the interferon-β gene with tightly linked promoter and enhancer activities
Source: Nucleic Acids Res. 2014 Oct 27;42(20):12537–54. doi: 10.1093/nar/gku1018 (PMC4227751; doi:10.1093/nar/gku1018)
Supplement: SUPPLEMENTARY DATA [file supp_42_20_12537__index.html]

A novel virus-inducible enhancer of the interferon-β gene with tightly linked promoter and enhancer activities — A novel virus-inducible enhancer of the interferon-β gene with tightly linked promoter and enhancer activities — SUPPLEMENTARY DATA 

# A novel virus-inducible enhancer of the *interferon-β* gene with tightly linked promoter and enhancer activities

## SUPPLEMENTARY DATA

**Files in this Data Supplement:**

- SUPPLEMENTARY DATA
- SUPPLEMENTARY DATA
