# Supplementary material for: A novel virus-inducible enhancer of the interferon-β gene with tightly linked promoter and enhancer activities
Source: Nucleic Acids Res. 2014 Oct 27;42(20):12537–54. doi: 10.1093/nar/gku1018 (PMC4227751; doi:10.1093/nar/gku1018)
Supplement: SUPPLEMENTARY DATA [file supp_gku1018_nar-02205-x-2014-File011.docx]

**Table S1.** Primers used in this study are described in the Excel Worksheet, “**Table S1 – Primers Used.xls**”. **Sheet “Cloning Primers”.** Primers used for cloning purposes are listed, including our name for the primer, the primer sequence, and the specific use of that primer. For amplification primers, added restriction sites are indicated in lower case. We then provide a reference list of construct names, and the nature of the experiments in which those constructs were used. **Sheet “3C Primers”.** Primers used for 3C are listed, including the genomic coordinates, the name for the primer, the primer sequence, and their relationship to any results discussed in the study. **Sheet “ChIP Primers”.** Primers used to detect transcription factor enrichment by ChIP are listed, including our name for the primer, the primer sequence, its use in the manuscript. **Sheet “Recombineering Oligos”.** Oligonucleotides used for recombineering the BAC CTD2104N16 are listed.

| Bac Clone IDs |
| --- |
| CTD 2292C18 |
| CTD 2104N16 |
| CTD 2503E5 |
| RP11 272C14 |
| RP11 319I22 |
| RP11 615P15 |
| RP11 758N4 |
| RP11 956G20 |
| RP11 666G3 |

**Table S2.** Clone IDs for the Bacterical Artificial Chromosomes used to generate the 3C random-ligation control library.

**Figure S1.** L2 promoter and enhancer activity is unresponsive to IFNβ treatment. **A.** IFNβ-responsiveness of L2 promoter activity was determined for both orientations. All constructs were analyzed in three independent experiments (n=3). **B.** IFNβ-responsiveness of L2 enhancer activity was determined for both orientations. All constructs were analyzed in three independent experiments (n=3). **A. & B**. Error bars indicate Standard Error. We tested for “significance” by looking for ≥2-fold differences between two samples, with unpaired T-tests to discern the statistical significance. A single asterisk, “*”, indicates both a ≥2-fold difference a p < 0.05. A double asterisk, “**”, indicates a ≥2-fold difference with p<0.01. The letters “ns” mean “not significant, and indicate either a ≤2-fold difference or p>.05.
